# On Hydrophobia

**Published:** 1809-03

**Authors:** 


*The Attention of the Legislature being at present directed to the Extermination of* RABIES, *the following Paper, communicated by* MR. TAUNTON, *from some MSS. in his Possession, cannot be indifferent to our Readers.*

RABIES CANINA, or, as it is more commonly called, Hydrophobia, the subject of the following dissertation, is a disease as little understood, yet as serious in its consequences, quences, and dreadful in its effects, as any with which the human body is affected.

It may be defined a painful and difficult state of deglutition, attended with great anxiety and horror of countenance, with occasional convulsive paroxysms; and these the consequence of the bite of a mad animal.

*History of the Disease.*

The symptoms take place at very irregular and uncertain intervals of time after the bite, having been known to occur as early as the third week, and as late as nine or twelve months; but for the most part the commencement of the disease may be placed at four or six weeks from the time of the accident. In most cases, the first symptom is a painful and uneasy sensation in the part where the bite was inflicted; but this is not to be considered as a constant or invariable occurrence.

Among the earliest appearances are to be ranked languor, depression of spirits, timidity, disturbed sleep, frightful dreams, sighing, and loss of appetite; sometimes with nausea, weight at the stomach, and rigor.

In a short time the unhappy object becones extremely sensible to all external impressions; the sense of touch, of hearing and seeing, being more or less affected in different cases. Upon attempting to swallow the smallest quantity either of solids or fluids, but especially the latter, although frequently excited to it by thirst, the greatest agitation and horror are produced, with an apparently strong convulsive affection of the pharynx and œsophagus, difficulty of breathing, tremor, great anxiety and impatience, and remarkable quickness of circulation.

The patient is not so much agitated by the sight of solids or fluids in the early state of the disease, as is commonly imagined; watery liquors being sometimes carried to the mouth with fortitude and composure; but, immediately on touching the lips, are rejected with a violent and frightful agitation, the mind being at that time more particularly conscious of the inability of swallowing.

Solids are, notwithstanding, in some cases, got down, even in the advanced stage of the disease, but never without pain and agitation, being thrust into the mouth in a peculiar hurried and greedy manner, and invariably exciting or increasing the convulsions, which constitute so formidable a part of the disease.

After these symptoms have continued twelve, eighteen, or twenty-four hours (the progress being somewhat different ent in different cases), the disorder puts on the most distressing and melancholy appearance; the convulsive attacks, which were before excited chiefly by the attempt to swallow, now occur spontaneously every ten or fifteen minutes, the whole body being so violently agitated as to require several assistants to support the patient. The countenance is wild, the eyes red and staring, and large drops of sweat pour from the head and face.

In the intervals of these paroxysms, the miserable sufferer becomes somewhat composed, complains of an uneasy sensation across the breast, and also in the throat, often ascribing it to wind, and wondering he is not able to discharge it, so as to obtain relief.

The secretion of saliva is now much increased, but of such a thick viscid quality, that the patient is obliged to exert considerable force to discharge it from his mouth. The manner of doing this, and the frequency with which it is repeated, joined to the peculiar anxious state of the countenance already mentioned, so strongly characterize the disease, that the most superficial observer, having seen one case of it, can scarcely afterwards be at a loss to distinguish another. As the disease draws towards a conclusion, the intellectual faculties, which had before remained wonderfully perfect, give way, the patient being affected with delirium of the fiercest and most unmanageable kind, especially during the paroxysms of convulsion, which become so frequent as scarcely to have any interval.

The strength is at length exhausted; the pulse is extremely small, weak, quick, and intermitting; cold clammy sweats supervene; the countenance is somewhat livid and frightfully distorted; and in this state a general return of convulsion puts an end to one of the most melancholy and affecting scenes that the human mind can well form an idea of. This fatal termination happens most commonly about the end of the third day from the first attack, though it has sometimes occurred as early as the second, and at other times as late as the fourth day.

No anatomical examination that has hitherto been made on this subject, seems to have thrown any light upon it. Different parts of the fauces, pharynx, and œsophagus have been frequently found slightly inflamed, probably owing to the exertions to which these parts are subjected in the course of the disease. The lungs have also been generally found distended with blood, and in some cases the vessels of the brain likewise; both of which may be supposed to depend on the irregular action of the heart.

The

The only disease with which this is likely to be confounded is tetanus; the painful and difficult deglutition, with the convulsive paroxysms, being common to both; but the continued stiffness of the jaws, or the spasm of the muscles by which the jaws are kept fixed, which is essential to tetanus, will at once lead to a satisfactory distinction, independent of the circumstances of infection, which we always annex to hydrophobia.

With regard to the theory of this disease, there are several important questions which naturally present themselves for consideration, each requiring a separate and perhaps extensive discussion, viz. To what is the origin of the affection to be ascribed? To what animals is it confined? Is any country exempted from it, and in what climates is it most frequent? Is the infection confined to the saliva? Is absorption necessary to the production of the disease? At how late a period is the destruction of the part on which the bite was inflicted, effectual in preventing the disorder? Are the symptoms accompanied by a state of increased excitement, or by debility?

The limits of this paper will not permit me to enlarge on these interesting topics; but we may shortly observe, that, whatever may be the origin of the infection, it does not appear to be confined to any particular class of animals, or any particular country or climate; that, with regard to absorption as necessary to the production of the disease, it is difficult to form a decided opinion; for while on the one hand we have the analogy of other poisons, as that of lues venerea, in favour of absorption, we must observe on the other, that this disease bears a great resemblance to tetanus, where there is not the most distant suspicion of any thing absorbed to account for the mischief; and further, that the lymphatic glands in the course of absorption have never, as far as I know, been found diseased.

Could this point be settled, we should have less difficulty in determining the next, namely, the period at which the destruction of the part bitten would be effectual in preventing the occurrence of the disease; for, were it clearly shown to be produced by absorption, we should be inclined to think that the operation would be successful any time before the commencement of the pain in the part mentioned in the history.

As to the question whether the hydrophobia be a disorder accompanied by increased excitement or debility, it is necessary to say, that, by increased excitement, I mean not only a greater frequency of action in the heart and arteries, but of strength likewise. In some cases of this disease, disease, the symptoms seem to have indicated such a condition; as for example, the rapid and apparently strong state of circulation, and the fierce and unmanageable delirium. Bleeding has seemed to be strongly indicated from these circumstances, and has accordingly had a full and fair trial; but the effects have by no means tended to confirm the idea on which the practice was founded. The delirium and frequency of circulation have rather, on the contrary, been increased by it. It is not unlikely, when we take a view of the action of the other poisons, that there may be a difference in different cases; thus, the small-pox is sometimes attended with strong inflammatory symptoms; at other times with great debility; and this difference not depending on any variety in the poison itself, but on the state of the constitution and other accidental circumstances. I am not able to determine how far the analogy will apply to hydrophobia. That it is sometimes connected with debility cannot be doubted, having occurred in delicate children, in whom the pulse has been weak throughout the whole of the disease. This weakness, too, must necessarily be increased by the unhappy state of deglutition, which precludes all nourishment by the mouth. There are several other circumstances also, which are favourable to the idea of debility as connected with this complaint: thus, it is well known that some of the most violent convulsive disorders are attended with great debility, and the only method of removing them effectually, is by strengthening the constitution. Again, a fierce and very unmanageable state of delirium occurs in some cases of low fever, where every other symptom points out weakness, and the free use of wine at such times has produced the happiest effects. Although then it is far from my intention to propose debility as explanatory of the symptoms of hydrophobia, yet it appears to be that condition of the body which most generally accompanies it, and which should not be lost sight of in the treatment.

### *Method of Treatment.*

I have no hesitation in affirming, that there is no well-marked case of this disorder in which a cure has been obtained after the symptoms have made their appearance; and even with regard to the prevention, that there is no method of treatment (that of removing the part excepted) which can in any degree be depended on.

The disorder has repeatedly occurred after the fullest trial of the Ormskirk and other boasted specifics. And no favourable favourable conclusion can be drawn frome those instances in which patients having taken such remedies have escaped. First, because many bites are inflicted by animals which are not diseased, but only supposed to be so. Secondly, If the animal be decidedly mad, all of those on whom the bite is inflicted are not the subjects of the disease, some of them escaping independently of any medicine.

After the symptoms have made their appearance, there are some remedies which appear to have had so full a trial, that their exhibition should be totally laid aside in future. Of these are the Ormskirk medicine, musk, mercurials, bleeding, warm bath, and opium; and therefore, in conducting the treatment hereafter, I would propose in the first place, that we should seek for a specific among those articles of the materia medica which are known to exert strong effects upon the body. Among the metallic preparations, I would more particularly recommend a trial of those of lead, copper, zinc, and lastly of arsenic. Among the vegetables, tobacco, cicuta, aconite, henbane, &c. Several remedies of this description may be administered at the same time.

But while we are endeavouring in this way to find out a specific for the poison of hydrophobia, I would not neglect other objects, which appear to be of consequence, and which do not interfere with it. Thus, I should endeavour to administer frequent clysters composed of broth, milk, and other nutritious articles. Various antispasmodics may be combined and employed in the same form, for it is in vain to expect that the patient can swallow so frequently as would be necessary to the fair trial of such remedies. Camphor, asafœtida, castor, æther, &c. may all be combined in the form of clyster, and injected every second or third hour. When these different plans have been tried, if the disease should still baffle our endeavours, let us not continue tame witnesses of so melancholy a spectacle, but proceed to methods which no other situation could justify; I mean that of injecting into the blood vessels various active remedies, having previously tried upon animals (as some kind of guide) in what quantities they can be received into circulation without fatal effects. If these should be found unsuccessful, to expose the patient for a certain length of time to one or other of the mephitic gases.

